# Functional characterization in *Caenorhabditis elegans *of transmembrane worm-human orthologs

**DOI:** 10.1186/1471-2164-5-85

**Published:** 2004-11-08

**Authors:** Anna Henricson, Erik LL Sonnhammer, David L Baillie, Ana Vaz Gomes

**Affiliations:** 1 Center for Genomics and Bioinformatics, Karolinska Institutet, Stockholm, Sweden; 2 Department of Molecular Biology and Biochemistry, Simon Fraser University, Burnaby, Canada

## Abstract

**Background:**

The complete genome sequences for human and the nematode *Caenorhabditis elegans *offer an opportunity to learn more about human gene function through functional characterization of orthologs in the worm. Based on a previous genome-wide analysis of worm-human orthologous transmembrane proteins, we selected seventeen genes to explore experimentally in *C. elegans*. These genes were selected on the basis that they all have high confidence candidate human orthologs and that their function is unknown. We first analyzed their phylogeny, membrane topology and domain organization. Then gene functions were studied experimentally in the worm by using RNA interference and transcriptional *gfp *reporter gene fusions.

**Results:**

The experiments gave functional insights for twelve of the genes studied. For example, C36B1.12, the worm ortholog of three presenilin-like genes, was almost exclusively expressed in head neurons, suggesting an ancient conserved role important to neuronal function. We propose a new transmembrane topology for the presenilin-like protein family. *sft-4*, the worm ortholog of surfeit locus gene Surf-4, proved to be an essential gene required for development during the larval stages of the worm. R155.1, whose human ortholog is entirely uncharacterized, was implicated in body size control and other developmental processes.

**Conclusions:**

By combining bioinformatics and *C. elegans *experiments on orthologs, we provide functional insights on twelve previously uncharacterized human genes.

## Background

The nematode *Caenorhabditis elegans *has been used as a simple model for understanding animal biology for nearly four decades. After the sequencing of entire genomes from several metazoans, we are now in an excellent position to take a gene-centric approach to the worm as a model organism. A majority of human genes have homologs in *C. elegans*. In a comparative proteomics study, 83% of the worm proteome was found to have human homologous genes [1]. Only 11% or less contains nematode specific genes. This makes the worm a suitable model organism for delineating human gene function [2-4].

In a previous study, all transmembrane protein families in the *C. elegans *genome were classified and the human orthologs identified [5]. Predicted proteins with two or more membrane domains were clustered and for each cluster a multiple alignment was created. From the alignments, HMMs (Hidden Markov Models) were built and subsequently used to search for mammalian homologs. The consensus of nine different phylogenetic methods and BLAST were used to assign orthology. This resulted in a total of 174 worm-human orthology assignments with a high confidence.

Orthologs are sequences that arose from a common ancestor gene and were separated by a speciation event [6]. Identification of orthologs is important, since they might share functionality. In closely related species, such as human and mouse, orthologs are normally trivial to find. However, when comparing distantly related species, e.g. human and worm, this is no longer the case because the similarity levels overall are low. Instead, one needs to rely on sophisticated phylogenetic reconstruction techniques to infer whether two genes stem from a node that corresponds to a speciation split or to a duplication event within a lineage. Close orthologs are likely to have the same biological role in the two organisms. Distant orthologs on the other hand, are less likely to have the same phenotypical role, but may have the same role in the corresponding pathway. Consequently, by studying true *C. elegans *orthologs to human genes experimentally in the worm, one can potentially learn more about the gene function also in humans. Depending on whether duplication(s) have occurred in one or both lineages since the speciation event, orthologs can form one-to-one, one-to-many or many-to-many relationships.

Paralogs arise from a duplication event. A common scenario when genes are duplicated is that one of the gene copies is under negative selective pressure and therefore retains the function of the ancestor. The other copy might then be more free to evolve a new function different from the ancestral function. This is the reason why paralogs in different species are less likely to share functionality compared to orthologs. Paralogs can be divided into two subtypes – outparalogs and inparalogs [7]. Outparalogs are paralogs that evolved by gene duplications that happened before the speciation event and therefore they do not form orthologous relationships. Inparalogs, on the other hand, form co-orthologous relationships, since they are paralogs that evolved by gene duplications that happened after the speciation event.

Here we present an initial functional characterization in *C. elegans *of seventeen genes. The criteria for selecting these genes were that they are high confidence candidate orthologs to human genes [5] and that their function is unknown. They are all predicted to encode transmembrane proteins, which imply that they could constitute as yet unknown receptors, channels or transporters playing important roles in various biological processes in multicellular organisms. We are particularly interested in studying those genes that might have a neuronal function. The phylogeny, membrane topology and domain organization were analyzed. Gene function was explored experimentally in the worm by means of RNA interference induced knock-down phenotypes and gene expression patterns.

## Results

### Membrane topology predictions

**Figure 1:**
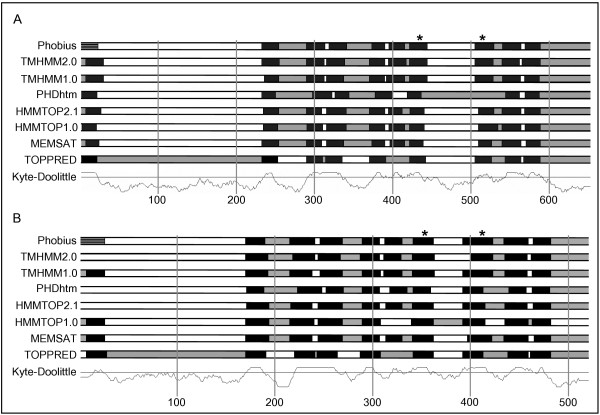
Output from SFINX for **(A) ***C. elegans *protein C36B1.12 and **(B) **one of its assigned human orthologs Q8TCT8. The overall membrane topology of the two proteins is very similar. The consensus from the different topology predictors is nine transmembrane (TM) regions, a N-terminal signal peptide, and a >150 amino acids non-cytoplasmic N-terminal region. Conserved aspartic acid residues are marked with an asterisk (residues 433 and 516 for C36B1.12; residues 351 and 412 for Q8TCT8). Phobius is the only program used that predicts both TM regions and N-terminal signal peptides. The other programs are designed to only detect TM regions, and therefore, they commonly mistake the signal peptide for a TM region. Numbers on the horizontal axis indicate amino acid positions. Color coding: black = TM region, white = non-cytoplasmic, gray = cytoplasmic, striped = N-terminal signal peptide predicted by Phobius.

The consensus of nine different methods was used to predict membrane topologies for the putative *C. elegans *proteins, and two different methods were used to predict signal peptides (see Methods for details). Each predicting method has some margin of error; therefore the consensus from several different predictors is more likely to give a better estimate of the true topology. Results were viewed using the SFINX tool [8,9], an example of output can be seen in Fig. 1. The number of transmembrane (TM) regions ranges between six and ten (except for one of the splice variants of R155.1), with a majority of proteins having six or seven TM regions (see Table 1). Such proteins are likely to be receptors, channels, or transporters. One case, however, (C36B1.12) is likely to be an intramembrane protease.

**Table 1:** Description of *C. elegans *– human orthologs. The Swiss-Prot accession numbers are given for the worm sequences and their human orthologs. TM: the number of transmembrane regions predicted using the consensus of nine different methods in the SFINX tool. Bootstrap support (%) is given for the inferred speciation node in the phylogenetic tree constructed using PHYLOWIN with PAM distances. Identity (%): sequence percentage identity from the Blastp output between the *C. elegans *gene and the nearest human ortholog. Four of the worm genes are predicted to have two splice variants (a and b). However, there are only small differences in the protein sequences between the two variants, except for R155.1. Splice variant R155.1b is predicted to have a truncation of more than one hundred amino acids in the N-terminus compared to R155.1a. The PF03062 domain (MBOAT) is still present in both splice variants. The protein sequence for R155.1a was used in the phylogenetic analysis.

***C. elegans *orthologs**	**TM**	**Pfam-A domains**	**Human orthologs**	**Bootstrap support (%)**	**Identity (%)**	**Nearest human ortholog with putative function**

**C30H6.2 **(Q9XVR4)	6	PF02535 (ZIP)	Q9H6T8, Q9NXC4, Q96NN4	57	50	Q9H6T8: SLC39A4, involved in intestinal absorption of zinc.

**T11F9.2a **(Q8I4G0), **T11F9.2b **(Q22395)	7	PF02535 (ZIP)	Q15043, Q96SM9, Q9C0K1, Q96BB3	91	30	Q9C0K1: BIGM103, involved in intracellular zinc retention and accumulation.

**H13N06.5 **(Q9XTQ7)	9	PF02535 (ZIP)	Q92504	98	52	Transport of zinc out of ER^2 ^and other intracellular stores.

**T28F3.3**^1 ^(Q9XUC4)	7*	PF02535 (ZIP)	Q92504	79	38	Transport of zinc out of ER^2 ^and other intracellular stores.

**T01D3.5 **(Q9XVJ5)	8	PF02535 (ZIP)	Q9NUM3	99	38	Function unknown.

**F40F9.1a **(Q8MQ56), **F40F9.1b **(Q8MQ55)	7	PF01027 (UPF0005)	Q9BWQ8, Q969X1	70	39	Q9BWQ8: Lifeguard protein, protects cells from Fas-mediated cell death.

**F40F9.2 **(Q20241)	7	PF01027 (UPF0005)	Q9BWQ8, Q969X1	70	40	Q9BWQ8: Lifeguard protein, protects cells from Fas-mediated cell death.

**F08F1.7 **(O17388)	9*	PF02990 (EMP70)	Q99805	100	62	Endosomal integral membrane protein.

**ZK858.6a **(Q94422)	9	PF02990 (EMP70)	Q92544	100	53	Function unknown.
**ZK858.6b **(Q7YTF9)	9*					

**F14F3.3 **(Q19468)	9	PF03062 (MBOAT)	Q96N66, Q99908	100	27	Q99908: BB1 protein, malignant cell expression-enhanced gene.

**R155.1a **(O01925)	8	PF03062 (MBOAT)	Q92980	100	32	Function unknown.
**R155.1b **(Q86DC4)	4					

**C36B1.12 **(Q93346)	9*	PF04258 (Peptidase_A22B)	Q8TCT7, Q8TCT8, Q8IUH8	100	30	Q8TCT7: Presenilin-like protein, may act as intra-membrane protease.

***sft-4 ***(Q18864)	7	PF02077 (SURF4)	O15260	100	55	Surfeit locus protein 4, probable ER^2 ^integral membrane protein.

**D2013.10 **(O62126)	6	None	Q15055	98	53	Function unknown.

**T04A8.12 **(Q22141)	6	None	Q9UHJ9	50	35	FRAG1 (FGFR (fibroblast growth factor receptor) activating gene 1).

**Y6B3B.10 **(Q9XWE9)	6	PF03798 (LAG1)	P27544	99	37	Function unknown.

**ZK721.1 **(Q9GYF0)	10*	None	Q9NXL6, Q9Y357	97	34	Function unknown.

^1 ^T28F3.3 was initially included because of strong similarity to Q92504; however, the phylogenetic analysis showed that it is probably an outparalog to the human gene.

^2 ^ER = endoplasmic reticulum.

* predicted to have a N-terminal signal peptide.

### Phylogenetic analysis

**Figure 2:**
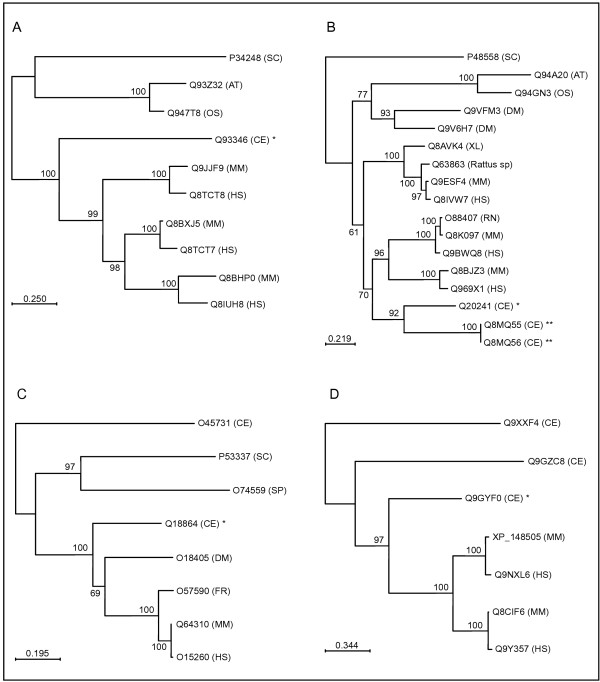
Phylogenetic trees for genes **(A) **C36B1.12 (Q93346), **(B) **F40F9.1a (Q8MQ56), F40F9.1b (Q8MQ55) and F40F9.2 (Q20241), **(C) ***sft-4 *(Q18864), and **(D) **ZK721.1 (Q9GYF0). The trees were constructed using PHYLOWIN with neighbor-joining method and PAM distances. 500 bootstrap replicates were run. All gene identifiers are Swiss-Prot accession numbers, except in (D) where XP_148505 is the NCBI accession number. The *C. elegans *genes studied here are marked with asterisks. F40F9.1 is predicted to have two splice variants; however, the putative proteins have the same length and only differ in the two most C-terminal amino acids. Species abbreviations: *Arabidopsis thaliana *(AT), *Caenorhabditis elegans *(CE), *Drosophila melanogaster *(DM), *Fugu rubripes *(FR), *Homo sapiens *(HS), *Mus musculus *(MM), *Oryza sativa *(OS), *Rattus norvegicus *(RN), *Saccharomyces cerevisiae *(SC), *Schizosaccharomyces pombe *(SP), *Xenopus laevis *(XL).

The results from the phylogenetic analysis are presented in Table 1. The previous orthology assignments are still valid [5], although for some *C. elegans *genes, additional human orthologs have emerged from the sequencing efforts. At present, 29% (5 of 17) of the worm genes have one-to-many ortholog relationship with human genes, which means that there has probably been an expansion in the human lineage. This is the case for C36B1.12 (Q93346) and ZK721.1 (Q9GYF0) (see Fig. 2A and 2D, respectively). 53% (9 of 17) showed a one-to-one relationship with its human ortholog, for example *sft-4 *(Q18864) (see Fig. 2C). Two of the worm genes, F40F9.1 (Q8MQ55, Q8MQ56) and F40F9.2 (Q20241), seem to have a many-to-many relationship (see Fig. 2B). The phylogenetic tree in Fig. 2B show somewhat inconclusive support for where the speciation event could have taken place. It is possible that the human gene Q8IVW7 is also an ortholog to the two worm genes. To investigate the ortholog relationship further, Orthostrapper was used [10]. Orthostrapper analyzes a set of bootstrap trees instead of the optimal tree for orthologs. The algorithm detects orthologous relations between two (groups of) species. The frequency of orthology assignments in the bootstrap trees can be interpreted as a confidence value for the possible orthology of two proteins. Orthology assignments in the optimal phylogenetic tree that might be incorrect can be identified by their low ortholog bootstrap value. This makes it possible to resolve complicated many-to-many orthologous relationships. When analyzing the multiple alignment for the phylogenetic tree in Fig. 2B using Orthostrapper, the results showed a stronger support for the human genes Q9BWQ8 and Q969X1 to be the orthologs compared to Q8IVW7 (65% vs. 23%). It seems that the orthologous relationship in this particular case is complicated to elucidate. Still, Q9BWQ8 and Q969X1 are the best candidate human orthologs for the *C. elegans *genes F40F9.1 and F40F9.2.

H13N06.5 and T28F3.3 both show sequence similarity to the human gene Q92504. However, the phylogenetic tree and results from Orthostrapper (data not shown), suggest that H13N06.5 is the putative ortholog to Q92504, whereas T28F3.3 may be an outparalog.

The bootstrap support for the speciation node between T04A8.12 and its human ortholog Q9UHJ9 barely made the cutoff of 50% when PAM distances was used. With observed divergence and Poisson correction as distance methods, the bootstrap support improved to 99% and 67%, respectively. When analyzing the phylogenetic tree using Orthostrappper, there was a very strong support for this orthology assignment (94%). Therefore, we conclude that T04A8.12 is probably the ortholog to Q9UHJ9.

C30H6.2 has three potential human orthologs, Q9H6T8, Q9NXC4 and Q96NN4. The bootstrap support for the speciation node with PAM distances was 57%. This improved to 87% and 88% with observed divergence and Poisson correction, respectively. Orthostrapper results showed a strong support for Q9H6T8 and Q9NXC4 as orthologs to C30H6.2 (84%), whereas the support for Q96NN4 was weaker (57%). Considering these results, we believe that all three human genes are orthologs to the worm gene; however, the ortholog relationship seems to be weaker between Q96NN4 and C30H6.2.

### Putative domain assignments

The domain organization of the predicted proteins was analyzed using the Pfam database [11,12] (see Table 1). Conclusions about possible functions cannot be drawn from the mere presence of a putative domain, although it can give some indication.

Five of the proteins (C30H6.2, T11F9.2, H13N06.5, T28F3.3 and T01D3.5) may have a PF02535 domain, which is annotated as being a ZIP domain. The ZIP family is believed to include zinc and other metal transporters. The ZIP proteins have been classified into four groups based on sequence conservation [13]; the ZIP subfamily I and II, the gufA subfamily and the LIV-1 subfamily (also called the LZT subfamily). The ZIP I subfamily contains mostly plant and yeast sequences; however, it also includes T01D3.5 and its putative orthologs in *Drosophila melanogaster*, mouse and human (Q9V4C6, Q8BFU1 and Q9NUM3, respectively). The other four worm genes appear to belong to the LIV-1 subfamily. This subfamily has a unique metalloprotease motif that raises the possibility that they might have protease activity [14]. Within the LIV-1 subfamily there is a subgroup called the KE4 group, to which H13N06.5 and its human ortholog hKE4 (Q92504) belong.

C36B1.12 was predicted to have a PF04258 domain, a probable signal peptide peptidase (SPP) domain. SPP catalyzes intramembrane proteolysis of some signal peptides after they have been cleaved from a preprotein. This processing by SPP is related to protein cleavage by presenilins. Homologs to SPP are divided into five subfamilies based on phylogenetic analysis (subfamily SPP and subfamilies SPPL1-4, for SPP like) [15]. C36B1.12 and its putative human orthologs Q8TCT7, Q8TCT8 and Q8IUH8 belong to the SPPL2 subfamily. The members of subfamilies SPPL1-4 only show homology to SPP in the C-terminal half of the protein and in the N-terminus there is substantial variation. This suggests that the C-terminal part may constitute the proteolytic subdomain, whereas the N-terminus defines the specific function of the respective proteins.

F40F9.1 and F40F9.2 seem to have a PF01027 domain (UPF0005), which is an uncharacterized protein family. Both F08F1.7 and ZK858.6 may belong to the PF02990 domain family (EMP70). Proteins in this family might be located to endosomal membranes [16]. F14F3.3 and R155.1 were predicted to have a PF03062 domain, which is annotated as a MBOAT (Membrane bound O-acyl transferases) domain. Biochemically characterized proteins of this group encode enzymes that transfer organic acids onto hydroxyl groups of membrane-embedded targets [17]. SFT-4 most likely has a PF02077 (SURF4) domain. Members of this family are believed to encode integral membrane proteins located to the endoplasmic reticulum [18]. A PF03798 domain (LAG1) was found in Y6B3B.10. This domain is associated with longevity in yeast (Jiang et al. 1998). Three of the seventeen putative proteins (D2013.10, T04A8.12 and ZK721.1) do not match to any Pfam-A domain.

### RNA interference studies

Out of the seventeen genes studied, *sft-4 *and R155.1 exhibited phenotypes when both the N2 (wildtype) and the RNAi sensitive *rrf-3(pk1426) *II [19,20] strains were subjected to RNAi by feeding (see Table 2). The phenotypes were enhanced with strain *rrf-3*, although the Dpy (dumpy) phenotype seen for R155.1 was still low penetrant and relatively weak. The Lva (larval arrest) observed for *sft-4 *occurred at larval stages L2–L3 and there was an almost complete penetrance with the sensitive strain. The RNAi phenotypes for both genes were detected at all temperatures, although, for *sft-4 *they were more severe at higher temperatures. The positive results were verified with RNAi by injection in strain N2.

**Table 2:** RNAi phenotype and major tissues of gene expression for *C. elegans *orthologs. Abbreviations: *RNAi phenotype: *clear (Clr), dumpy (Dpy), larval arrest (Lva), ruptured (Rup), sterile (Ste), wildtype (WT). *Gene expression: *body wall muscle (bwm), commissures (c), excretory system (exc), gonad (g), hypodermis (h), hypodermal seam cells (hs), intestine (i), neuronal (n), pharyngeal muscle (phm), rectal epithelial cells (re), spermatheca (s), vulva (v), ventral nerve cord (vnc). A limitation when extrachromosomal array transgenes are used is that expression in the germ line is not possible to evaluate. No transgenic lines could be generated for T11F9.2, H13N06.5 and T04A8.12. Possible reasons for this could be that the injected DNA concentration was too low or that the sequence was toxic. In either case, the extrachromosomal array formed may not have been sufficiently large to be inheritable [46]. The transgenic lines for ZK858.6 and F14F3.3 showed no expression of *gfp*. This might be caused by conditional gene expression, germline silencing or absence of the promoter::*gfp *fusion from the inheritable extrachromosomal array [46].

***C. elegans *orthologs**	**RNAi phenotype**	**Major tissues of gene expression**
C30H6.2	WT	h, phm
T11F9.2a, T11F9.2b	WT	No transgenic line
H13N06.5	WT	No transgenic line
T28F3.3	WT	h, i, n, v, vnc
T01D3.5	WT	hs
F40F9.1a, F40F9.1b	WT	bwm, c, h, n, phm, vnc
F40F9.2	WT	exc, n, phm
F08F1.7	WT	h, n, phm, re, s, v
ZK858.6a, ZK858.6b	WT	No expression
F14F3.3	WT	No expression
R155.1a, R155.1b	Dpy	bwm, h, i, phm
C36B1.12	WT	i, n
*sft-4*	Clr, Lva, Rup, Ste	bwm, h, i, n, phm, v
D2013.10	WT	bwm, h, i, n, s, v
T04A8.12	WT	No transgenic line
Y6B3B.10	WT	phm
ZK721.1	WT	bwm, g, h, i, n, phm, s, v

### Analysis of gene expression

Transcriptional fusions with *gfp *were established for fourteen genes and the resulting gene expression was analyzed. The results are presented in Table 2. Because the arrays are extrachromosomal and not integrated; mosaic patterns of expression were observed. Also, germ line expression could not be analyzed, due to germ line silencing. For 18% (3 of 17) of the genes no transgenic lines could be established despite several attempts, and out of the lines established, 14% (2 of 14) showed no expression. This could be due to several reasons (see Discussion). In half of the transgenic lines established, expression was found in more than three different tissues. The most prevalent major tissues of expression were hypodermis (9 of 14 transgenic lines), nervous system and pharyngeal muscle (8 of 14) and intestine (6 of 14).

**Figure 3:**
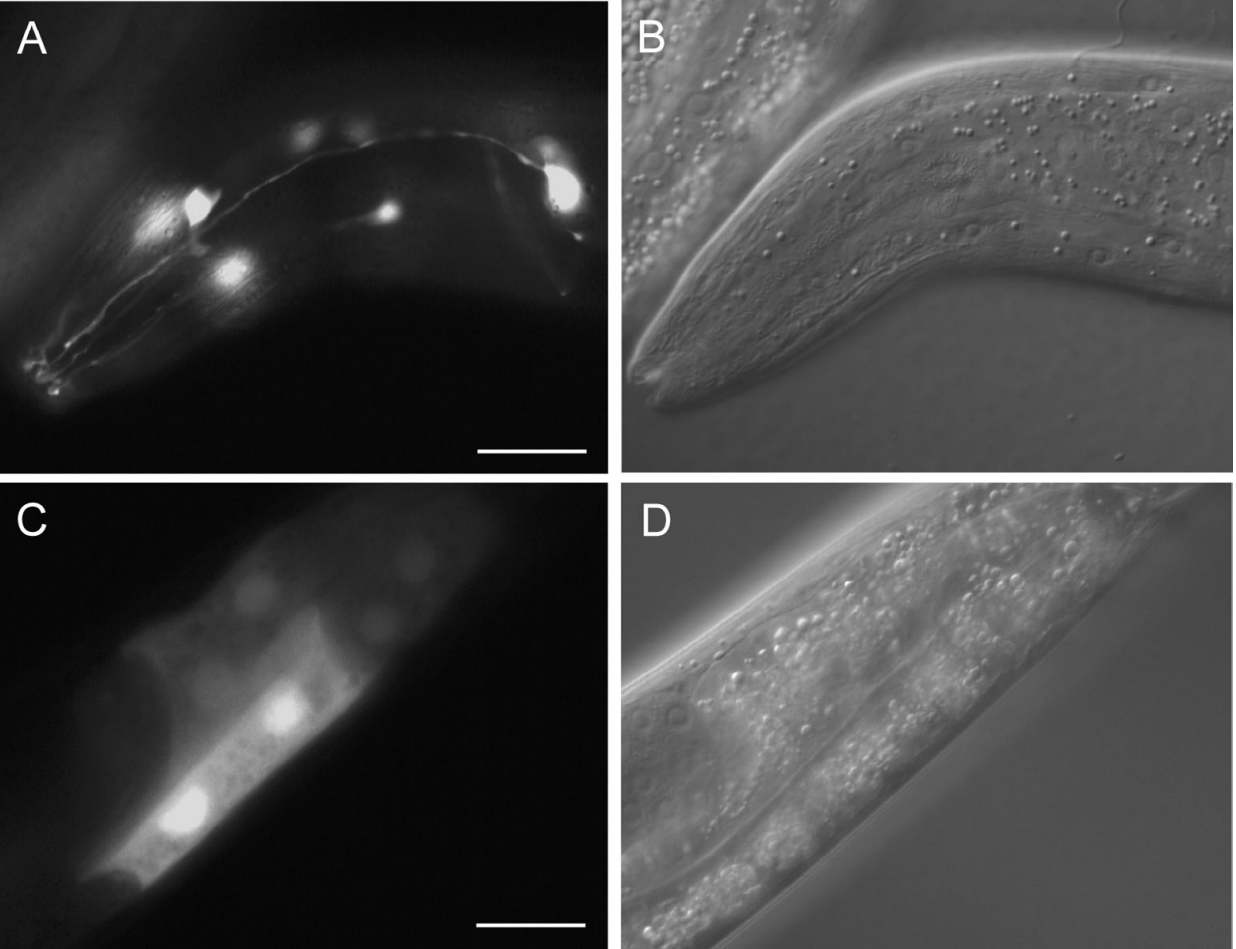
Major tissues of expression for C36B1.12. **(A) **Fluorescence micrograph of an L4 larvae hermaphrodite carrying a transcriptional fusion between *gfp *and a putative promoter of C36B1.12 expressed in neurons in the head. **(C) **Fluorescence micrograph of a young adult hermaphrodite carrying the same construct expressed in intestine. The observed intestinal expression is mostly located to posterior intestinal nuclei and is more prominent in younger worms. **(B and D) **Corresponding DIC images. Scale bars, 20 μm.

**Figure 4:**
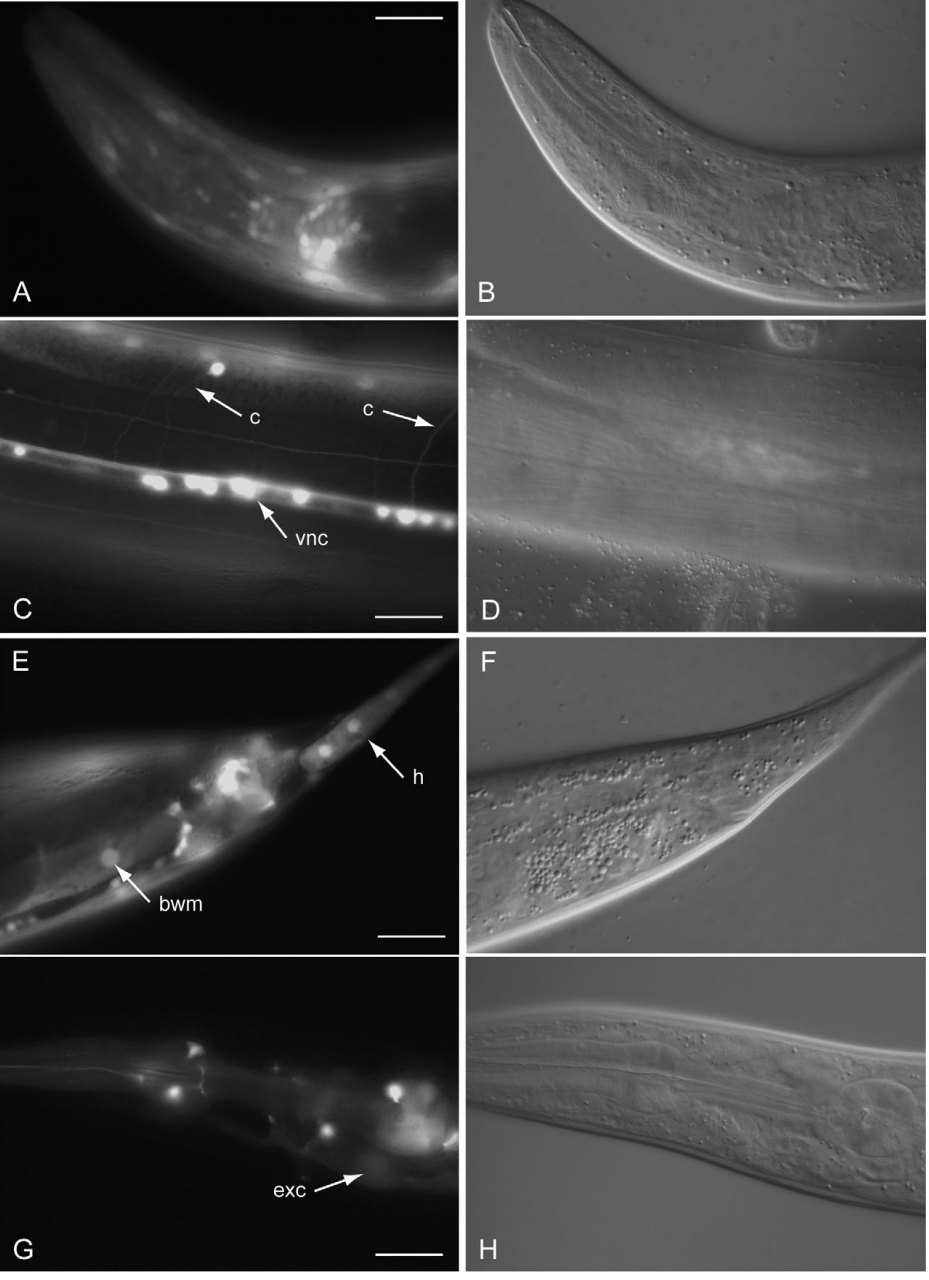
Major tissues of expression for F40F9.1 and F40F9.2. Fluorescence micrographs of an adult hermaphrodite carrying a transcriptional fusion between *gfp *and a putative promoter of F40F9.1 expressed in **(A) **neurons and pharyngeal muscle, **(C) **commissures (c) and the ventral nerve cord (vnc), and **(E) **body wall muscle (bwm) and hypodermis (h). **(G) **Fluorescence micrograph of an L4 hermaphrodite carrying a transcriptional fusion between *gfp *and a putative promoter of F40F9.2 expressed in the excretory system (exc), neurons, and pharyngeal muscle. **(B, D, F, and H) **Corresponding DIC images. Scale bars, 20 μm.

**Figure 5:**
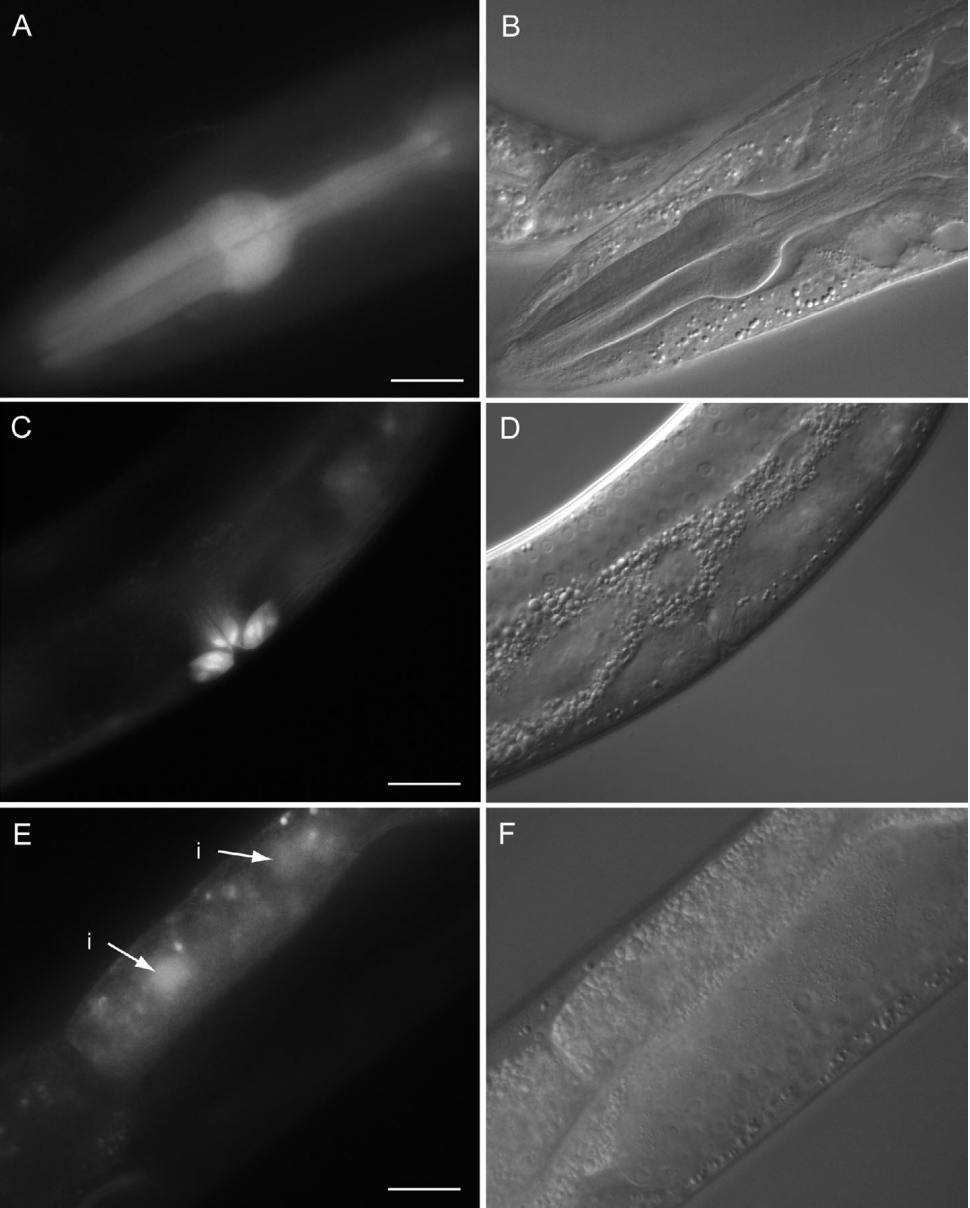
Major tissues of expression for *sft-4*. Fluorescence micrographs of an adult hermaphrodite carrying a transcriptional fusion between *gfp *and a putative promoter of *sft-4 *expressed in **(A) **pharyngeal muscle, **(C) **vulva and **(E) **intestinal nuclei (i). The intestine shows some unspecific autofluorescence, but there is also specific expression in the intestinal nuclei. Expression in body wall muscle, hypodermis and neurons is not shown. **(B, D, and F) **Corresponding DIC images. Scale bars, 20 μm.

**Figure 6:**
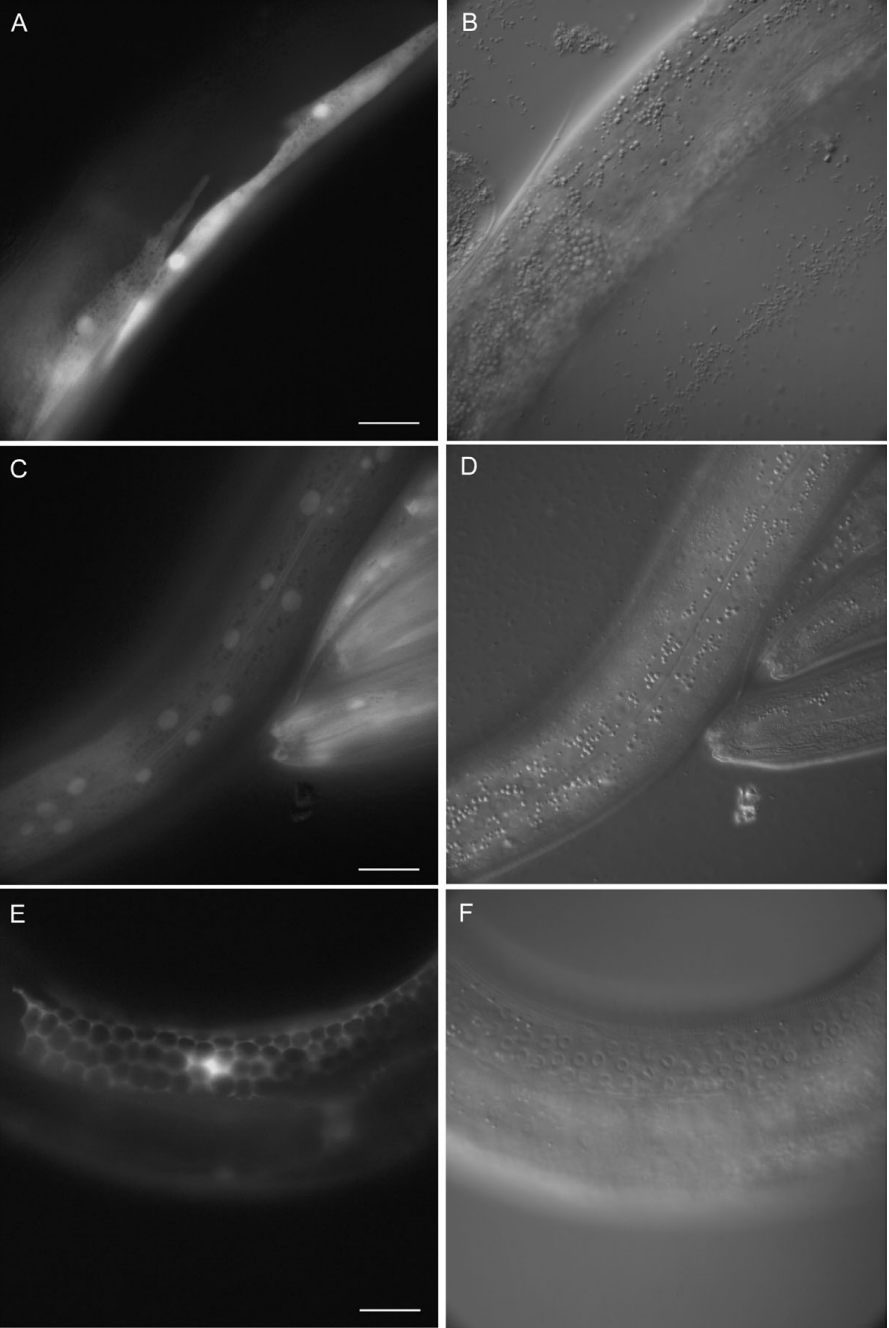
Major tissues of expression for ZK721.1. Fluorescence micrographs of an adult hermaphrodite carrying a transcriptional fusion between *gfp *and a putative promoter of ZK721.1 expressed in **(A) **body wall muscle, **(C) **hypodermis and **(E) **gonad. Gene expression in hypodermis is weaker compared to expression in other tissues. Expression in intestine, neurons, pharyngeal muscle, spermatheca, and vulva is not shown. **(B, D and F) **Corresponding DIC images. Scale bars, 20 μm.

Examples of gene expression patterns observed are presented in Fig. 3, 4, 5, 6. C36B1.12 shows expression restricted to head neurons and intestine (see Fig. 3). The intestinal expression was stronger during larval stages compared to the adult stage, and it was predominantly located to posterior intestinal nuclei. F40F9.1 and F40F9.2 demonstrate some overlapping expression in nervous system and pharyngeal muscle (see Fig. 4A,4G); however, F40F9.1 appear to be more widely expressed in the nervous system with expression in more neuronal cell bodies and in commissures and ventral nerve cord (see Fig. 4C). Expression of F40F9.1 is also located to body wall muscle and hypodermal cells in the tail (see Fig. 4E). F40F9.2 is also expressed in the excretory system, although it is weaker compared to the other tissues (see Fig. 4G). Widespread expression patterns were observed for *sft-4 *and ZK721.1 both during larval and adult stages (see Fig. 5 and 6, respectively). For *sft-4 *it was highly mosaic with pharyngeal muscle as the most consistent tissue of expression.

### Putative function assignments

#### C36B1.12

The three putative human orthologs (Q8TCT7, Q8TCT8 and Q8IUH8) to the *C. elegans *gene C36B1.12 are thought to be presenilin-like (PSL) proteins (also called PSH proteins for presenilin homologs). Presenilins are an important group of proteases acting in the nervous system. Abnormal proteolytic cleavage may result in accumulation of pathogenic insoluble proteins, implied in e.g. Alzheimer's disease. We have shown that C36B1.12 is probably expressed in head neurons and intestine; however, the intestinal expression might be ectopic (see Discussion for details), which would imply that the gene is exclusively expressed in neurons. This suggests that the three human orthologs may also encode neuronal functions.

The membrane topology of human proteins belonging to the presenilin-like family has been analyzed previously. Q8IUH8 was predicted to have seven transmembrane (TM) segments and a cytoplasmic C-terminus [21], and the same was predicted for HM13_HUMAN (Swissprot: Q8TCT9) [15]. However, it should be noted that although the number of TM segments of these predictions is the same, the topologies are in fact different. The fourth segment in the Q8IUH8 prediction is missing from the HM13_HUMAN prediction, and the C-terminal segment in the HM13_HUMAN prediction is missing from the Q8IUH8 prediction. This means that the four C-terminal TM segments are not in register between the predictions, and consequently the loops are on opposite sides. This includes the loop between the putatively catalytic aspartic acid residues also present in presenilins that was predicted cytoplasmic by Ponting et al., and non-cytoplasmic by Weihofen et al. Because of the TM segment disagreement, these aspartic acid residues were predicted to be located in TM5 and TM6 in the Ponting et al. prediction, but in TM4 and TM5 in the Weihofen et al. prediction.

Merging these two proposed topologies by accepting all TM segments predicted by one or the other study would yield a topology with nine TM segments. Our own analysis of the proteins in question using the SFINX tool [8,9] provides strong support for this topology, with the C-terminus in the cytoplasm (data not shown). We therefore propose that both previous TM topologies had incorrectly left out one TM segment, which would correspond to segments 4 and 9 in the 9-TM segment model.

We further analyzed the other members of this family with SFINX [8,9], and consistently found a 9-TM topology model with C-terminus in the cytoplasm. The conserved aspartic acid residues would be located in TM6 and TM7. As an example, the SFINX output for C36B1.12 and one of its human orthologs Q8TCT8 is shown in Fig. 1. The overall topology is very similar between the putative worm protein and all of its orthologs in both human and mouse, as well as the other presenilin-like proteins. One difference, however, is that C36B1.12 and two of its human (Q8TCT8 and Q8IUH8) and mouse orthologs (Q9JJF9 and Q8BHP0) are predicted to have a N-terminal signal peptide, a feature that seems to be missing from the other presenilin-like proteins.

#### F40F9.1 and F40F9.2

F40F9.1 and F40F9.2 are 48% identical to each other on the protein sequence level and they also appear to have similar membrane topologies. They are close in the genome (<100 bp apart), but on opposite strands. It has been shown that genes closer than 500 bp on opposite strands are likely to have a shared control region [22], which means that these two genes might be coexpressed. The expression patterns observed are indeed overlapping, although not to a full extent (see Table 2). One of the human orthologs (Q9BWQ8) identified has been shown to protect cells from Fas-mediated cell death [23], suggesting that F40F9.1 and F40F9.2 might be involved in apoptosis.

#### sft-4

The *sft-4 *gene (C54H2.5) is highly conserved throughout evolution with orthologs in both vertebrates and non-vertebrates. All orthologous relationships are one-to-one with a high bootstrap support. The tree in Fig. 2C indicates that there exists a worm homolog (O45731) to *sft-4*. O45731 (T02E1.7) was found to be 33% identical to *sft-4 *on the protein sequence level and it was also predicted to have a PF02077 (SURF4) domain. However, our RNAi screen indicates that there is no or little functional redundancy between the two genes, since *sft-4 *has a very strong RNAi phenotype, showing an almost complete larval arrest at stages L2–L3. The RNAi phenotype for T02E1.7 is wildtype according to previous studies [24]. Our gene expression analysis revealed a wide spread expression of the reporter construct for *sft-4 *(see Table 2). Taken together, these data suggests that *sft-4 *may play an essential role during development acting in many tissues.

#### ZK721.1

ZK721.1 is most probably orthologous to the human genes Q9NXL6 and Q9Y357 (CGI-40 protein). The CGI-40 protein was found in a screen where novel human genes evolutionary conserved in *C. elegans *were identified [1]. The function of both CGI-40 and Q9NXL6 is unknown. ZK721.1 has several worm homologs, one of which is *sid-1 *(Q9GZC8). SID-1 has been identified as a protein that is required for systemic RNAi [25,26]. It was predicted to have eleven transmembrane (TM) regions and some of them have been experimentally verified [27]. The high number of TM regions suggests that SID-1 forms a channel. Double stranded RNA is thought to diffuse through this channel, leading to spreading of the RNA and hence, a systemic RNAi effect. This idea is also supported by the fact that no homolog of *sid-1 *has been found in *Drosophila*, which can explain the observed absence of systemic RNAi in this organism [28,29]. Our analysis of ZK721.1 predicts that it has ten TM regions, which makes it a likely candidate for forming a channel. The phylogenetic tree indicates that ZK721.1 is the best candidate ortholog to human genes Q9NXL6 and Q9Y357, whereas *sid-1 *is a probable outparalog to the human genes (see Fig. 2D). The tree also supports the previous finding that there is no homolog to ZK721.1 or *sid-1 *in the *Drosophila *genome. We have observed a wildtype RNAi phenotype for ZK721.1, which is consistent with results from other studies [30,31]. Further analysis might reveal if ZK721.1 also is involved in systemic RNAi or if it has some other function. Four additional genes required for systemic RNAi have been reported (*rsd-2*, *-3*, *-4 *and *-6*) [26], but none of them map to locus ZK721.1.

#### C30H6.2, H13N06.5, T01D3.5, T11F9.2 and T28F3.3

SLC39A4 (Q9H6T8) was identified as one of three possible human orthologs to C30H6.2. The human gene has been implicated in the rare inherited condition acrodermatitis enteropathica, which results from a defect in the absorption of zinc [32]. It is believed that SLC39A4 might encode a zinc transporter responsible for intestinal absorption of zinc. Therefore, C30H6.2 may also be a zinc/metal transporter.

The predicted human ortholog (Q92504) to H13N06.5 in *C. elegans *has been shown to be a zinc transporter localized to intracellular membranes [33]. Q92504 probably transports zinc out of the endoplasmic reticulum and other intracellular stores. The *Drosophila *ortholog to H13N06.5 is *Catsup *(*Catecholamines up*, Q9V3A4), which encodes a negative regulator of tyrosine hydroxylase (TH) activity [34]. TH is a rate-limiting enzyme for production of dopamine in the brain. The *Arabidopsis thaliana *gene *IAR1 *(Q9M647) is also an ortholog to H13N06.5. It is proposed to be involved in auxin metabolism or response [35]. Interestingly, the mouse ortholog (Q31125) to H13N06.5 and *IAR1 *was shown to functionally substitute for the *Arabidopsis *gene. These data indicate that there is functional conservation among these orthologs and it is likely that H13N06.5, and possibly also T28F3.3, could play similar roles in the corresponding pathways in the worm.

BIGM103 (Q9C0K1) was identified as one of four candidate human orthologs to T11F9.2. The human gene was found to be induced during the infection and inflammatory response. It was also shown to play a role in intracellular zinc ion accumulation and retention [36]. Consequently, it is possible that T11F9.2 might be an integral membrane zinc/metal transporter.

The human ortholog to T01D3.5 has no known putative function. We observed a wildtype RNAi phenotype for T01D3.5 as well as for the other four PF02535 (ZIP) domain containing putative proteins (C30H6.2, H13N06.5, T11F9.2 and T28F3.3). This indicates that there might be some functional redundancy between these genes. Considering the information available, it is conceivable that T01D3.5 may also be a zinc/metal transporter.

#### F08F1.7, T04A8.12 and ZK858.6

F08F1.7 and ZK858.6 show 47% identity on the protein sequence level and they have similar membrane topologies with a large N-terminal non-cytoplasmic region and nine transmembrane regions in the C-terminal part. F08F1.7 and one of the splice variants of ZK858.6 were predicted to have a N-terminal signal peptide. The phylogenetic analysis indicated that the human gene p76 (Q99805) is the ortholog to F08F1.7 and it appears to localize to endosomes [37]. The function of the probable human ortholog (Q92544) to ZK858.6 is unknown.

Both F08F1.7 and ZK858.6 are predicted to be in operons, as is T04A8.12 [38]. F08F1.7 is probably in an operon with *tth-1 *(F08F1.8, O17389). TTH-1 is likely to belong to the PF01290 domain family (thymosin beta-4), which includes actin-binding proteins, implicating a possible role in cytoskeleton organization. ZK858.6 is predicted to be in an operon with ZK858.5 (Q94421) and ZK858.7 (Q94416). A PF05154 (TM2) domain with unknown function is likely to be present in ZK858.5. ZK858.7 might have a PF04189 domain (eIF3gamma; eukaryotic initiation factor 3, gamma subunit), suggesting that it could be involved in translation. T04A8.12 may be in an operon with T04A8.11 (Q22140) and T04A8.13 (Q22142). T04A8.11 might be a ribosomal protein, since it appears to have a PF00252 (ribosomal L16) domain. T04A8.13 was predicted to have a PF00646 (F-box) domain, which is known for forming structural complexes with other proteins. There are no matching Pfam-A domains for T04A8.12. The best candidate human ortholog to T04A8.12 is FRAG1 (fibroblast growth factor receptor activating gene 1, Q9UHJ9). FRAG1 seems to be ubiquitously expressed in adult human tissues and it has also been detected in several human tumor cell lines [39]. Our results from the gene expression analysis demonstrated that F08F1.7 is probably expressed in several tissues in *C. elegans*, suggesting an important biological role. Previous studies have shown that the human ortholog p76 (Q99805) is ubiquitously expressed [37]. There was no expression observed for ZK858.6 and for gene T04A8.12, we failed to generate a transgenic line. All three genes exhibited a wildtype RNAi phenotype. For F08F1.7 and ZK858.6, the wildtype phenotype could possibly be explained by functional redundancy between the two genes and a third *C. elegans *EMP70 domain containing protein Y41D4A.4 (Q95Y24). We found that Y41D4A.4 is homologous to F08F1.7 and ZK858.6, and most likely an ortholog to the human gene Q9HD45. Taken together, these findings point to that F08F1.7, ZK858.6 and T04A8.12 might play fundamental biological roles, and that they may be involved in processes such as cellular organization (F08F1.7) and translation (ZK858.6 and T04A8.12).

#### F14F3.3 and R155.1

The human genes Q99908 (BB1) and Q96N66 are probable orthologs to F14F3.3. BB1 has been shown to be overexpressed in breast and bladder carcinoma [40], suggesting that it might have a role in tumor progression. The function of Q96N66 is unknown.

The likely *Drosophila *ortholog to R155.1 is *Nessy *(Q9XYV9), a putative Hox gene target [41], indicating a possible developmental role. The Dpy (dumpy) RNAi phenotype detected for R155.1 could be due to some developmental/body size regulatory error in possibly the hypodermis and/or body wall muscle; tissues in which the gene is expressed according to our analysis. The best human ortholog candidate (Q92980) to R155.1 has not yet been functionally characterized.

#### Y6B3B.10

Y6B3B.10 is most probably orthologous to the human gene P27544 and they both seem to belong to the PF03798 (LAG1) domain family. LAG1 is a longevity gene that was cloned from yeast [42]. Members of the LAG1 family are thought to be involved in determining lifespan. However, the phylogenetic tree revealed that Y6B3B.10 and its human and mouse ortholog (P27545) form a tight cluster in the tree, separated from the other LAG1 domain containing proteins, indicating that they may have evolved a slightly different function. Y6B3B.10 showed a wildtype RNAi phenotype and it appears to have an expression restricted to the pharyngeal muscle.

#### D2013.10

D2013.10 is orthologous to Q15055 (human), Q8K1A5 (mouse) and Q9VX39 (*Drosophila*). Neither of these genes has any putative function assigned to them and they have no matching Pfam-A domains. D2013.10 is expressed in several tissues in *C. elegans *and it exhibits a wildtype RNAi phenotype.

## Discussion

This study illustrates how bioinformatic and experimental analysis can be combined to elucidate putative gene function. We have predicted worm-human orthologs and performed an initial functional characterization of the worm genes. Since orthologs are likely to have the same biological function, a better understanding of the function of the human genes can be accomplished through analysis in *C. elegans*. The genes explored in this study were selected from a previous study [5] and they are all predicted to encode transmembrane proteins. Such proteins are attractive to study since many interesting receptors, channels, transporters and signaling proteins are found among them, making them likely to be involved in important regulatory processes in multicellular organisms.

The number of transmembrane (TM) regions predicted for each protein, is similar to the number predicted for each cluster of putative TM proteins from the former study (± 1 TM region) [5]. For the putative proteins H13N06.5, F14F3.3 and ZK721.1, however, the difference is larger (+2-3 TM regions). This divergence could be due to Remm and Sonnhammer having performed predictions on a cluster and not on individual genes. In addition, they used only the program TMHMM [43] for analyzing membrane topology. TMHMM, when using default settings, may miss weak TM regions, leading to a possible underestimation of the number of TM segments. A better estimate of the true topology can be achieved through the use of several different prediction programs. In this study, we used the consensus of nine different methods provided by the SFINX tool [8,9] to assign membrane topology.

We observed an RNAi phenotype for 11.8% (2 of 17) of the genes when using both strains N2 (wildtype) and *rrf-3 *(RNAi sensitive), respectively. This is in agreement with previous experiments, where 10.3% (N2) and 12.8% (*rrf-3*) phenotypes have been detected [30,44]. The RNAi phenotypes for *sft-4 *are consistent with previous results [30,31]. However, the two groups have reported non-overlapping phenotypes, but in this screen we have observed all of them. The Lva (larval arrest) phenotype has also been reported from the genome wide screen using strain *rrf-3 *[44]. The Dpy (dumpy) phenotype for R155.1 has not been reported before. The gene was downregulated using RNAi by injection in a screen of chromosome III [45] and the phenotype was found to be wildtype. However, the focus of that analysis was to identify genes involved in cell division and therefore only a few post-embryonic phenotypes were scored. The Dpy phenotype observed is also low penetrant and relatively weak and could therefore be missed. Furthermore, differences in results from RNAi screens have been shown to exist. A 10–30% difference between experiments done in both different and in the same laboratories has been reported [44].

When analyzing expression patterns using transcriptional reporter fusions, one issue of concern is whether the pattern observed is the expression pattern of the native gene or not. Ectopic or lack of expression can occur if the putative promoter used does not include all the regulatory elements. Expression in several different cell types in the pharynx and in the posterior intestinal cells of young animals has been attributed to the use of incomplete promoters [46]. Another limitation when using extrachromosomal arrays is that analysis of expression in the germ line is not possible, due to germ line silencing.

The putative promoter used in the transcriptional fusion for C36B1.12 is only 1 kb, due to the presence of an upstream gene. Therefore, it is possible that the intestinal expression seen predominantly in young worms and mostly located to posterior intestinal nuclei, is an artifact of the use of an incomplete promoter region [46]. If this is the case, C36B1.12 might be expressed exclusively in neurons in the head (see Fig. 3). This finding provides support to the idea that C36B1.12 and its three human orthologs encode neuronal functions. A possible consequence of this could be that they act in a fashion analogous to presenilin, or even that they could be involved in β-amyloid precursor protein processing. It would be interesting to study their role in nervous system development and function, and whether they are linked to neurological disorders

The transcriptional fusion for T28F3.3 also showed a similar intestinal expression, apart from the specific expression in neurons in the head, ventral nerve cord, vulva and a weak expression in hypodermis. The putative promoter region used is only 0.8 kb, due to the presence of an upstream gene. Thus, the intestinal expression seen for T28F3.3 may once again be related to the use of an incomplete promoter region [46].

Two of the transcriptional fusions (for the genes F14F3.3 and ZK858.6) showed no expression of the reporter gene. This is unlikely due to the use of an incomplete promoter region, since the upstream region included was 2.9 kb and 3.0 kb, respectively. Instead, it might be caused by conditional gene expression, germline silencing or absence of the promoter::*gfp *fusion from the inheritable extrachromosomal array [46]. For three of the genes in this study we failed to establish transgenic lines. Possible reasons for this could be that the injected DNA concentration was too low or that the sequence was toxic. In either case, the extrachromosomal array formed may not have been sufficiently large to be inheritable [46].

Out of the seventeen genes in this study, three are predicted to be in operons (18%). This is equivalent to the number of genes in the *C. elegans *genome that are believed to be in operons (15%). Whether *C. elegans *operons contain genes of related function or not is still unknown. There are, however, some indications that genes encoding proteins of fundamental biological importance might be clustered into operons. For example, genes for mitochondrial proteins have a strong tendency to be together in operons and also genes encoding splicing proteins [38].

## Conclusions

This study has shed some light upon the putative function of a few predicted worm-human orthologs. Our aim was to identify genes that could play a role in the nervous system and indeed we have been able to find eight genes that appear to be expressed in neurons. *C. elegans *is an excellent model organism for pursuing the functional characterization of these genes, considering its well mapped and relatively sophisticated nervous system. Investigating the function of orthologous proteins using a simple multicellular organism is a suitable approach for the possibility of learning more about the function of a gene not only in one species but also hopefully in several. This approach becomes even more valid as several genomes are being sequenced at the moment with additional ones already in the pipeline. With the enormous amount of data that these sequencing efforts are generating, it is very useful to be able to start delineating the gene function based on functional characterization of the ortholog in another species, before initiating studies in more complex organisms.

## Methods

### Membrane topology predictions

The membrane topology was predicted with nine different methods, and the SFINX tool [8,9], was used to display the results. Eight membrane topology predictors were used: Phobius [47], TMHMM2.0 [48], TMHMM1.0 [43], PHDhtm [49], HMMTOP2.1 [50], HMMTOP1.0 [51], MEMSAT [52] and TOPPRED [53]. In addition, a Kyte-Doolittle hydrophobicity curve [54] was constructed for each putative protein sequence. Transmembrane regions were considered positive if they were predicted by a majority of the methods, or by four methods and having a supporting Kyte-Doolittle hydrophobicity curve. Phobius also predicts N-terminal signal peptides. The signal peptides predicted by Phobius were verified with SignalP1.1 [55]. Each program was used with default settings.

### Databases

The Pfamseq database version 10.0 [56] was used for searching for homologous sequences. It is based on the Swiss-Prot 41.10 and SP-TrEMBL 23.15 databases. The Pfam database [11] version 11.0 [12] was used for domain assignments.

### Phylogenetic analysis

The Pfamseq database [56] was searched for homologs using the Blastp 2.2.5 program [57] with default settings and with the putative worm proteins as query. Multiple alignments of full-length sequences were created using POA [58] with default settings. Gappy sequences and columns (>50% gaps) and redundant sequences (>99% identical) were removed. The program PHYLOWIN [59] with tree building method neighbor-joining [60] and PAM distance was used for constructing phylogenetic trees. Trees were also built with observed divergence and Poisson correction as distance methods, however, the results from that analysis are only discussed for genes where there were major differences in bootstrap support. If available, a yeast sequence was used as an outgroup. A total of 500 bootstrap tests were run on trees to assess the significance of the branching order. Only bootstrap values ≥ 50% were considered positive.

### Domain assignments

Pfam-A domains were assigned using the Pfam database [11,12]. Pfam-B domains were not considered, since they are automatically generated and non-curated and therefore of lower quality.

### Nematode strains and culture conditions

Maintenance and handling of *C. elegans *strains were as previously described [61]. Strains used were CGC N2 (wildtype) and CGC NL2099 (*rrf-3(pk1426) *II) [19] (Caenorhabditis Genetics Center [62]). The *rrf-3 *mutant strain has an increased sensitivity to RNAi, also for neuronal genes [20], which otherwise are more refractory towards RNAi compared to other tissue types. Strain CB00907 (*dpy-5(e907) *I) was used for generating the transgenic lines [63].

### RNAi screening

#### Generation and cloning of PCR products

Total RNA extracted and purified from *C. elegans *using TRIzol (Invitrogen Life Technologies, Carlsbad, CA) was reverse transcribed using Reverse Transcription System (Promega, Madison, WI) and then PCR products were generated using Advantage™ 2 PCR Enzyme System (Clontech, Palo Alto, CA) with gene specific primers as well as primers for spliced leader 1 (SL1) and SL2 (Invitrogen Life Technologies): 95°C 60 s, 35 cycles of (95°C 30 s, 55°C 30 s, 68°C 4 min) followed by an additional extension at 68°C 4 min. See additional data file 1 for the primer sequences used for the RNAi studies. Products were ligated into linearized (XmaI) (New England Biolabs, Frankfurt am Main, Germany) and dephosphorylated L4440 vector (Fire Laboratory [64]) using Rapid DNA Ligation Kit (Roche, Mannheim, Germany) and transformed into JM109 *E. coli *bacterial strain (Promega). Colonies were screened using XmaI, correct colonies were grown in overnight cultures and DNA was extracted using QIAfilter Plasmid Kit (QIAGEN, Hilden, Germany). The vector with the insert was sequenced using ABI PRISM^® ^Big Dye™ Terminator Cycle Sequencing Ready Reaction Kits (Applied Biosystems, Foster City, CA).

#### RNAi by feeding

Strains N2 and *rrf-3 *were used for RNAi screening by feeding [30,65]. CGC [62] bacterial strain *E. coli *HT115(DE3) was transformed with the L4440 vector (Fire Laboratory [64]) containing the cloned gene fragment using standard methods. The vector contains an ampicillin (Amp) resistance and strain HT115 is tetracycline (Tet) resistant, so bacteria were selected on Amp (75 μg/ml) and Tet (12.5 μg/ml) plates. Single colonies were picked and grown in cultures of LB with Amp (60 μg/ml) and Tet (12.5 μg/ml) for 14–17 h. The bacterial solution was seeded onto NGM plates containing 1 mM IPTG and 25 μg/ml carbenicillin. Seeded plates were allowed to dry at room temperature. Eggs were prepared with standard bleaching method and transferred to the plates. N2 strain was incubated at 15, 20 and 25°C. *rrf-3 *was incubated only at 15°C and 20°C, since the strain has a temperature-sensitive decrease in broodsize [20]. The hatched worms and their progeny were scored for a number of different phenotypes [30,44]. The phenotypes assayed were: Adl (adult lethal), Bli (blistering of cuticle), Bmd (body morphology defect), Brd (low broodsize), Clr (clear), Dpy (dumpy), Egl (egg laying defect), Emb (embryonic lethal), Gro (slow post-embryonic growth), Him (high incidence of males), Lon (long body), Lva (larval arrest), Lvl (larval lethal), Mlt (molt defect), Muv (multivulva), Prz (paralyzed), Pvl (protruding vulva), Rol (roller), Rup (ruptured), Sck (sick), Sma (small), Ste (sterile), Stp (sterile progeny), Unc (uncoordinated). Emb was defined as greater than 10% dead embryos for N2 and greater than 30% dead embryos for *rrf-3*. Ste and Stp required a brood size of fewer than ten for N2 and fewer than five for *rrf-3*. Each postembryonic phenotype was required to be present among at least 10% of the analyzed worms. The experiment was ongoing for about 4 generations and the phenotypes were scored on a daily basis. A constant supply of transformed HT115 bacteria was ensured. The experiments were performed in duplicates at each temperature for each gene and worm strain. For the postembryonic phenotypes typically at least 20 worms per plate were scored. As a positive control the gene *unc-22 *("twitchin") was used (Fire Laboratory vector pPD34.09 [64]). Empty L4440 vector was used as negative control.

#### RNAi by injections

The L4440 vector (Fire Laboratory [64]) containing the cloned gene fragment was linearized in two separate reactions using restriction enzymes NcoI and XhoI (New England Biolabs, Frankfurt am Main, Germany), respectively. The reactions were purified and single stranded RNA was synthesized using T7 RNA polymerase (Promega, Madison, WI). The two reactions were mixed and annealing was performed to produce double stranded (ds) RNA, which was subsequently purified. The dsRNA was injected undiluted into twelve young adult N2 hermaphrodites for each gene. The injected worms were put on individual plates and split between three different incubation temperatures (15, 20 and 25°C). Phenotypes were scored for both the injected worms and two subsequent generations on a daily basis. Phenotypes scored and criteria for scoring were the same as for the RNAi by feeding of strain N2.

#### Generation of transgenic lines

The transgenic lines were constructed at the Baille Laboratory, Simon Fraser University, Canada [66]. Transcriptional expression constructs for gonadal injection were generated using fusion PCR, also known as "PCR-stitching" [67]. Typically, about 3 kb of genomic DNA sequence immediately upstream of the predicted ATG initiator site, was used as the putative promoter (see supplementary material for primer sequences). When an upstream gene was within the 3 kb, the size of the putative promoter was adjusted downwards. For genes in operons, the sequence upstream of the first gene in the operon was used. The putative promoter was fused with another DNA fragment containing *gfp *(green fluorescent protein) and *unc-54 *3'UTR amplified from vector pPD95.67 (Fire Laboratory [64]). See additional data file 1 for the primer sequences used for generating the fusion PCR products. The resulting fusion PCR product was injected without purification into the gonad of young adult hermaphrodites of strain CB00907 at a concentration of 10 ng/μl together with 100 ng/μl *dpy-5*(+) plasmid (pCeh361) in 1xTE buffer to generate an extrachromosomal array. Analysis of the expression patterns of the different transgenic lines was performed at the Vaz Gomes Laboratory, Karolinska Institutet, Sweden.

## Author's contributions

AH carried out the membrane topology predictions, phylogenetic analysis, domain assignments, RNAi studies and analysis of gene expression in the transgenic worm strains. ES participated in the membrane topology predictions, phylogenetic analysis and domain assignments. DB contributed in making the transgenic worm strains. AVG participated in the RNAi studies and analysis of gene expression. All authors read and approved the final manuscript.

## Supplementary Material

Additional File 1 Primer sequences used in RNAi and gene expression studies. Click here for file

## References

[B1] Lai CH, Chou CY, Ch'ang LY, Liu CS, Lin W (2000). Identification of novel human genes evolutionarily conserved in Caenorhabditis elegans by comparative proteomics. Genome Res.

[B2] Kenyon C (2001). A conserved regulatory system for aging. Cell.

[B3] O'Kane CJ (2003). Modelling human diseases in Drosophila and Caenorhabditis. Semin Cell Dev Biol.

[B4] Schulenburg H, Kurz CL, Ewbank JJ (2004). Evolution of the innate immune system: the worm perspective. Immunol Rev.

[B5] Remm M, Sonnhammer E (2000). Classification of transmembrane protein families in the Caenorhabditis elegans genome and identification of human orthologs. Genome Res.

[B6] Fitch WM (1970). Distinguishing homologous from analogous proteins. Syst Zool.

[B7] Sonnhammer EL, Koonin EV (2002). Orthology, paralogy and proposed classification for paralog subtypes. Trends Genet.

[B8] Sonnhammer EL, Wootton JC (2001). Integrated graphical analysis of protein sequence features predicted from sequence composition. Proteins.

[B9] SFINX. http://sfinx.cgb.ki.se/.

[B10] Storm CE, Sonnhammer EL (2002). Automated ortholog inference from phylogenetic trees and calculation of orthology reliability. Bioinformatics.

[B11] Bateman A, Birney E, Cerruti L, Durbin R, Etwiller L, Eddy SR, Griffiths-Jones S, Howe KL, Marshall M, Sonnhammer EL (2002). The Pfam protein families database. Nucleic Acids Res.

[B12] Pfam at the Sanger Institute, version 11.0. ftp://ftp.sanger.ac.uk/pub/databases/Pfam/old_releases/Pfam11.0/.

[B13] Gaither LA, Eide DJ (2001). Eukaryotic zinc transporters and their regulation. Biometals.

[B14] Taylor KM, Nicholson RI (2003). The LZT proteins; the LIV-1 subfamily of zinc transporters. Biochim Biophys Acta.

[B15] Weihofen A, Binns K, Lemberg MK, Ashman K, Martoglio B (2002). Identification of signal peptide peptidase, a presenilin-type aspartic protease. Science.

[B16] Chluba-de Tapia J, de Tapia M, Jaggin V, Eberle AN (1997). Cloning of a human multispanning membrane protein cDNA: evidence for a new protein family. Gene.

[B17] Hofmann K (2000). A superfamily of membrane-bound O-acyltransferases with implications for wnt signaling. Trends Biochem Sci.

[B18] Reeves JE, Fried M (1995). The surf-4 gene encodes a novel 30 kDa integral membrane protein. Mol Membr Biol.

[B19] Sijen T, Fleenor J, Simmer F, Thijssen KL, Parrish S, Timmons L, Plasterk RH, Fire A (2001). On the role of RNA amplification in dsRNA-triggered gene silencing. Cell.

[B20] Simmer F, Tijsterman M, Parrish S, Koushika SP, Nonet ML, Fire A, Ahringer J, Plasterk RH (2002). Loss of the putative RNA-directed RNA polymerase RRF-3 makes C. elegans hypersensitive to RNAi. Curr Biol.

[B21] Ponting CP, Hutton M, Nyborg A, Baker M, Jansen K, Golde TE (2002). Identification of a novel family of presenilin homologues. Hum Mol Genet.

[B22] Lercher MJ, Blumenthal T, Hurst LD (2003). Coexpression of neighboring genes in Caenorhabditis elegans is mostly due to operons and duplicate genes. Genome Res.

[B23] Somia NV, Schmitt MJ, Vetter DE, Van Antwerp D, Heinemann SF, Verma IM (1999). LFG: an anti-apoptotic gene that provides protection from Fas-mediated cell death. Proc Natl Acad Sci U S A.

[B24] Fraser AG, Kamath RS, Zipperlen P, Martinez-Campos M, Sohrmann M, Ahringer J (2000). Functional genomic analysis of C. elegans chromosome I by systematic RNA interference. Nature.

[B25] Winston WM, Molodowitch C, Hunter CP (2002). Systemic RNAi in C. elegans requires the putative transmembrane protein SID-1. Science.

[B26] Tijsterman M, May RC, Simmer F, Okihara KL, Plasterk RH (2004). Genes Required for Systemic RNA Interference in Caenorhabditis elegans. Curr Biol.

[B27] Feinberg EH, Hunter CP (2003). Transport of dsRNA into cells by the transmembrane protein SID-1. Science.

[B28] Piccin A, Salameh A, Benna C, Sandrelli F, Mazzotta G, Zordan M, Rosato E, Kyriacou CP, Costa R (2001). Efficient and heritable functional knock-out of an adult phenotype in Drosophila using a GAL4-driven hairpin RNA incorporating a heterologous spacer. Nucleic Acids Res.

[B29] Fortier E, Belote JM (2000). Temperature-dependent gene silencing by an expressed inverted repeat in Drosophila. Genesis.

[B30] Kamath RS, Fraser AG, Dong Y, Poulin G, Durbin R, Gotta M, Kanapin A, Le Bot N, Moreno S, Sohrmann M, Welchman DP, Zipperlen P, Ahringer J (2003). Systematic functional analysis of the Caenorhabditis elegans genome using RNAi. Nature.

[B31] Maeda I, Kohara Y, Yamamoto M, Sugimoto A (2001). Large-scale analysis of gene function in Caenorhabditis elegans by high-throughput RNAi. Curr Biol.

[B32] Wang K, Zhou B, Kuo YM, Zemansky J, Gitschier J (2002). A novel member of a zinc transporter family is defective in acrodermatitis enteropathica. Am J Hum Genet.

[B33] Taylor KM, Morgan HE, Johnson A, Nicholson RI (2004). Structure-function analysis of HKE4, a member of the new LIV-1 subfamily of zinc transporters. Biochem J.

[B34] Stathakis DG, Burton DY, McIvor WE, Krishnakumar S, Wright TR, O'Donnell JM (1999). The catecholamines up (Catsup) protein of Drosophila melanogaster functions as a negative regulator of tyrosine hydroxylase activity. Genetics.

[B35] Lasswell J, Rogg LE, Nelson DC, Rongey C, Bartel B (2000). Cloning and characterization of IAR1, a gene required for auxin conjugate sensitivity in Arabidopsis. Plant Cell.

[B36] Begum NA, Kobayashi M, Moriwaki Y, Matsumoto M, Toyoshima K, Seya T (2002). Mycobacterium bovis BCG cell wall and lipopolysaccharide induce a novel gene, BIGM103, encoding a 7-TM protein: identification of a new protein family having Zn-transporter and Zn-metalloprotease signatures. Genomics.

[B37] Schimmoller F, Diaz E, Muhlbauer B, Pfeffer SR (1998). Characterization of a 76 kDa endosomal, multispanning membrane protein that is highly conserved throughout evolution. Gene.

[B38] Blumenthal T, Evans D, Link CD, Guffanti A, Lawson D, Thierry-Mieg J, Thierry-Mieg D, Chiu WL, Duke K, Kiraly M, Kim SK (2002). A global analysis of Caenorhabditis elegans operons. Nature.

[B39] Lorenzi MV, Castagnino P, Aaronson DC, Lieb DC, Lee CC, Keck CL, Popescu NC, Miki T (1999). Human FRAG1 encodes a novel membrane-spanning protein that localizes to chromosome 11p15.5, a region of frequent loss of heterozygosity in cancer. Genomics.

[B40] Fukunaga-Johnson N, Lee SW, Liebert M, Grossman HB (1996). Molecular analysis of a gene, BB1, overexpressed in bladder and breast carcinoma. Anticancer Res.

[B41] Maurel-Zaffran C, Chauvet S, Jullien N, Miassod R, Pradel J, Aragnol D (1999). nessy, an evolutionary conserved gene controlled by Hox proteins during Drosophila embryogenesis. Mech Dev.

[B42] D'Mello NP, Childress AM, Franklin DS, Kale SP, Pinswasdi C, Jazwinski SM (1994). Cloning and characterization of LAG1, a longevity-assurance gene in yeast. J Biol Chem.

[B43] Sonnhammer EL, von Heijne G, Krogh A (1998). A hidden Markov model for predicting transmembrane helices in protein sequences. Proc Int Conf Intell Syst Mol Biol.

[B44] Simmer F, Moorman C, Van Der Linden AM, Kuijk E, Van Den Berghe PV, Kamath R, Fraser AG, Ahringer J, Plasterk RH (2003). Genome-Wide RNAi of C. elegans Using the Hypersensitive rrf-3 Strain Reveals Novel Gene Functions. PLoS Biol.

[B45] Gonczy P, Echeverri C, Oegema K, Coulson A, Jones SJ, Copley RR, Duperon J, Oegema J, Brehm M, Cassin E, Hannak E, Kirkham M, Pichler S, Flohrs K, Goessen A, Leidel S, Alleaume AM, Martin C, Ozlu N, Bork P, Hyman AA (2000). Functional genomic analysis of cell division in C. elegans using RNAi of genes on chromosome III. Nature.

[B46] Mello C, Fire A, Epstein HF, Shakes DC (1995). DNA transformation. In Caenorhabditis elegans: Modern biological analysis of an organism.

[B47] Kall L, Krogh A, Sonnhammer EL (2004). A combined transmembrane topology and signal peptide prediction method. J Mol Biol.

[B48] Krogh A, Larsson B, von Heijne G, Sonnhammer EL (2001). Predicting transmembrane protein topology with a hidden Markov model: application to complete genomes. J Mol Biol.

[B49] Rost B, Fariselli P, Casadio R (1996). Topology prediction for helical transmembrane proteins at 86% accuracy. Protein Sci.

[B50] Tusnady GE, Simon I (2001). The HMMTOP transmembrane topology prediction server. Bioinformatics.

[B51] Tusnady GE, Simon I (1998). Principles governing amino acid composition of integral membrane proteins: application to topology prediction. J Mol Biol.

[B52] Jones DT, Taylor WR, Thornton JM (1994). A model recognition approach to the prediction of all-helical membrane protein structure and topology. Biochemistry.

[B53] Claros MG, von Heijne G (1994). TopPred II: an improved software for membrane protein structure predictions. Comput Appl Biosci.

[B54] Kyte J, Doolittle RF (1982). A simple method for displaying the hydropathic character of a protein. J Mol Biol.

[B55] Nielsen H, Engelbrecht J, Brunak S, von Heijne G (1997). A neural network method for identification of prokaryotic and eukaryotic signal peptides and prediction of their cleavage sites. Int J Neural Syst.

[B56] Pfam at the Sanger Institute, version 10.0. ftp://ftp.sanger.ac.uk/pub/databases/Pfam/old_releases/Pfam10.0/.

[B57] Altschul SF, Madden TL, Schaffer AA, Zhang J, Zhang Z, Miller W, Lipman DJ (1997). Gapped BLAST and PSI-BLAST: a new generation of protein database search programs. Nucleic Acids Res.

[B58] Lee C, Grasso C, Sharlow MF (2002). Multiple sequence alignment using partial order graphs. Bioinformatics.

[B59] Galtier N, Gouy M, Gautier C (1996). SEAVIEW and PHYLO_WIN: two graphic tools for sequence alignment and molecular phylogeny. Comput Appl Biosci.

[B60] Saitou N, Nei M (1987). The neighbor-joining method: a new method for reconstructing phylogenetic trees. Mol Biol Evol.

[B61] Brenner S (1974). The genetics of Caenorhabditis elegans. Genetics.

[B62] Caenorhabditis Genetics Center. http://biosci.umn.edu/CGC/CGChomepage.htm.

[B63] Hodgkin J, Edgley M, Riddle DL, Albertson DG, Wood WB (1988). Appendix 4: Genetics. In The nematode Caenorhabditis elegans.

[B64] Fire Laboratory. ftp://www.ciwemb.edu/pub/FireLabInfo/.

[B65] Timmons L, Fire A (1998). Specific interference by ingested dsRNA. Nature.

[B66] McKay SJ, Johnsen R, Khattra J, Asano J, Baillie DL, Chan S, Dube N, Fang L, Goszczynski B, Ha E, Halfnight E, Hollebakken R, Huang P, Hung K, Jensen V, Jones SJM, Kai H, Li D, Mah A, Marra M, McGhee J, Newbury R, Pouzyrev A, Riddle DL, Sonnhammer E, Tian H, Tu D, Tyson JR, Vatcher G, Warner A, Wong K, Zhao Z, Moerman DG (2003). Gene expression profiling of cells, tissues and developmental stages of the nematode C. elegans. In Cold Spring Harbor symposium LXVIII: The genome of Homo sapiens.

[B67] Hobert O (2002). PCR fusion-based approach to create reporter gene constructs for expression analysis in transgenic C. elegans. Biotechniques.

